# Semisynthetic Pneumococcal Glycoconjugate Nanovaccine

**DOI:** 10.1021/acs.bioconjchem.3c00252

**Published:** 2023-09-11

**Authors:** Maruthi Prasanna, Rubén Varela Calvino, Annie Lambert, Maria Arista Romero, Sylvia Pujals, François Trottein, Emilie Camberlein, Cyrille Grandjean, Noemi Csaba

**Affiliations:** † Center for Research in Molecular Medicine and Chronic Diseases, Department of Pharmacology, Pharmacy and Pharmaceutical Technology, University of Santiago de Compostela, Santiago de Compostela 15706, Spain; ‡ Nantes Université, CNRS, Unité des Sciences Biologiques et des Biotechnologies (US2B), UMR 6286, Nantes F-44000, France; § Department of Biochemistry and Molecular Biology, University of Santiago de Compostela, Santiago de Compostela 15706, Spain; ∥ Univ. Lille, CNRS, INSERM, CHU Lille, Institut Pasteur de Lille, U1019—UMR 9017—CIIL—Center for Infection and Immunity of Lille, Lille F-59000, France; ⊥ Department of Biological Chemistry, Institute for Advanced Chemistry of Catalonia (IQAC-CSIC), Barcelona 08034, Spain

## Abstract

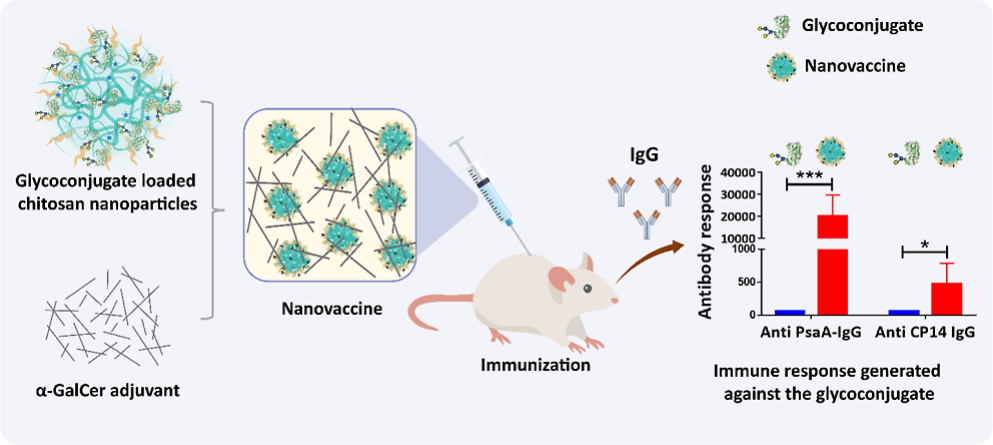

Pneumococcal conjugate vaccines offer an excellent safety profile and high protection against the serotypes comprised in the vaccine. However, inclusion of protein antigens from*Streptococcus pneumoniae*combined with potent adjuvants and a suitable delivery system are expected to both extend protection to serotype strains not represented in the formulation and stimulate a broader immune response, thus more effective in young children, elderly, and immunocompromised populations. Along this line, nanoparticle (NP) delivery systems can enhance the immunogenicity of antigens by protecting them from degradation and increasing their uptake by antigen-presenting cells, as well as offering co-delivery with adjuvants. We report herein the encapsulation of a semisynthetic glycoconjugate (GC) composed of a synthetic tetrasaccharide mimicking the*S. pneumoniae* serotype 14 capsular polysaccharide (CP14) linked to the Pneumococcal surface protein A (PsaA) using chitosan NPs (CNPs). These GC-loaded chitosan nanoparticles (GC-CNPs) were not toxic to human monocyte-derived dendritic cells (MoDCs), showed enhanced uptake, and displayed better immunostimulatory properties in comparison to the naked GC. A comparative study was carried out in mice to evaluate the immune response elicited by the glycoconjugate-administered subcutaneously (SC), where the GC-CNPs displayed 100-fold higher IgG response as compared with the group treated with nonencapsulated GC. Overall, the study demonstrates the potential of this chitosan-based nanovaccine for efficient delivery of glycoconjugate antigens.

## Introduction

*Streptococcus pneumoniae*is the leading cause of mortality in children under the age of 5 years. Pneumonia is responsible for 14% of all deaths in children under the age of five, killing 740,180 children in 2019. There are more than 96 serotypes of*S. pneumoniae* based on the diversity of capsular polysaccharides.^1^ The first vaccines employed in the prevention of pneumococcal infection are based on these capsular polysaccharides. The introduction of conjugate vaccines has significantly reduced the global burden of pneumococcal infections across many communities.^2^ Pneumococcal polysaccharide vaccines (PPSV23) and pneumococcal conjugate vaccines (PCV7 and PCV13) are now widely used in the clinic. Nonetheless, conjugate vaccines protect against limited serotypes whose polysaccharide components are incorporated in the vaccines. As a result, immunizing with the currently existing vaccines fails to offer protection against *S. pneumoniae*infections caused by those serotypes that are not included in the formulations. Polysaccharide or conjugate vaccines require complex manufacturing, purification, and dose optimization steps. In addition, the introduction of further serotypes in the formulations may result in an escalation of the vaccine costs. To circumvent these limitations, vaccines based on pneumococcal proteins have been investigated. Proteins like pneumococcal surface adhesin A (PsaA),^3−5^ pneumococcal surface protein A,^6^ pneumolysin,^7^ pneumococcal histidine triad D,^8^ and ATP-binding cassette transporter lipoprotein PiuA^9^ have been extensively studied as potential vaccine components active against pneumococcal infections as they are expected to provide Th17 cellular immunity.^10,11^ The combination of protein and sugar components has been proposed to induce an additive or synergistic effect.^12^ Along this line, we reported a semisynthetic glycoconjugate vaccine where PsaA plays a dual role both as an immunogen and as a carrier. PsaA was covalently linked to a synthetic tetrasaccharide (Pn14TS) to obtain a glycoconjugate (GC), characterized by a 5.4 Pn14TS/PsaA molar ratio on average.^13^ Pn14TS is a synthetic tetrasaccharide {Galβ(1 → 4)Glcβ(1 → 6)[Galβ(1 → 4)]GlcNAc} derived from the capsular polysaccharide of *S. pneumoniae* serotype 14, which is able to evoke an opsonophagocytic response.^14^ PsaA is a highly conserved, 37 kDa protein that is commonly present in all the 96 serotypes of *S. pneumoniae*.^15^ It is a member of the ATP-binding cassette protein that transports manganese, which is essential for virulence. Additionally, PsaA is surface-exposed, playing a significant role in the adhesion and colonization of *S. pneumoniae*.^15,16^ This makes PsaA an ideal candidate for vaccination against pneumococcal infections.^17,18^

While most subunit vaccines alone are poor immunogens that fail to elicit T- and B-cell responses, glycoconjugate vaccines can induce better immune protection. Nevertheless, their co-administration with an adjuvant like alum, cholera toxin,^19,20^ and α-galactosylceramide (α-GalCer)^21^ can further enhance the immune response. The α-GalCer adjuvant used in this study is a glycosphingolipid that can potentially activate the NKT cells^22^ and has been explored as an adjuvant in both systemic and mucosal immunizations.^23−25^ As a complementary strategy, the use of particulate carriers offers multiple benefits as a vaccine delivery system: (i) they tend to mimic the pathogens with their size, shape, and often by carrying the antigens at the surface; (ii) they can act as an adjuvant; (iii) they enhance the stability of antigens; and (iv) they reduce the need of administering multiple doses.^26−30^ Several particulate systems like polymeric nanoparticles (NPs),^31,32^ metallic NPs,^33^ liposomes,^34^ and microparticles^35^ have been explored for the delivery of pneumococcal antigens. Polymers like chitosan, alginate, protamine, dextran, hyaluronic acid, and poly(lactic-*co*-glycolic acid) have been investigated for protein/peptide^36^ or DNA-based vaccines.^37^ Among them, chitosan is a nontoxic, biocompatible, biodegradable, and mucoadhesive natural polymer, which is considered as an excellent biomaterial for the design of antigen carriers.^38^ As an immune adjuvant, chitosan is known to enhance humoral and cellular immunity.^39^ Despite its positive attributes, limitations such as burst release, poor mechanical properties, and stability in biological media pose a stiff challenge to its universal acceptability as a drug carrier.^40^ However, the previously mentioned positive features of CNPs dominate its shortcomings. Therefore, these properties fostered researchers to adopt chitosan nanoparticles (CNPs) as a carrier for vaccine delivery.

The preparation of CNPs involves a solvent-free and simple ionic gelation method,^36^ which makes them ideal for encapsulating delicate macromolecules like protein-based antigens. In this study, GC was encapsulated into CNPs to produce GC-CNPs, and the characterization of the resulting GC-CNPs was performed. The uptake of GC-CNPs by human dendritic cells (DCs) was studied *in vitro*. To further demonstrate the applicability of CNPs as a potential antigen carrier, their ability to induce the upregulation of costimulatory markers related to the DC stimulation was also evaluated. Finally, a comparison of GC and GC-CNPs was carried out *in vivo*, in order to determine the role of nanoencapsulation in enhancing the immunogenicity of the glycoconjugates.

## Results

### NP Preparation and Characterization

**Figure 1:**
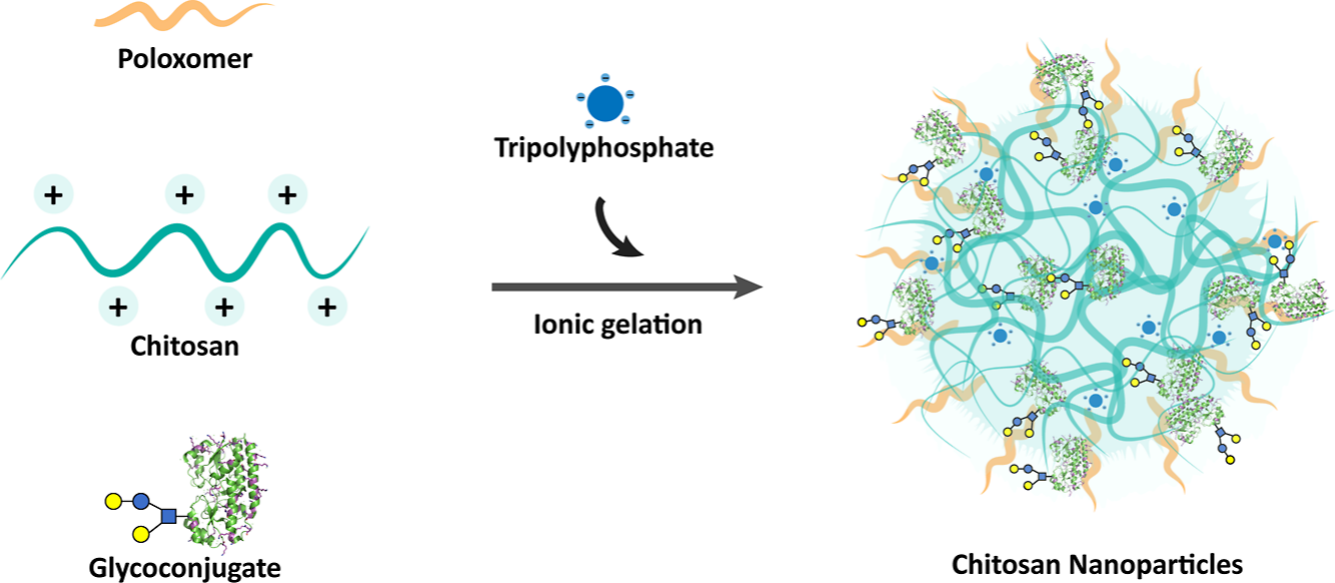
Schematic representation of CNP preparation by the ionic gelation method.

CNPs were prepared from chitosan (80–95% deacetylation, 30–400 kDa), poloxamer 188, and sodium tripolyphosphate in the presence or absence of GC^13^ by the ionic gelation technique that was previously developed by our group (Figure 1).^36^ The incorporation of poloxamer in the formulation and the use of glycerol bed during NP separation are attributed for its ability to maintain the stability of the NPs^41^ and improve the *in vivo* efficiency of the NPs.^42,43^

**Figure 2:**
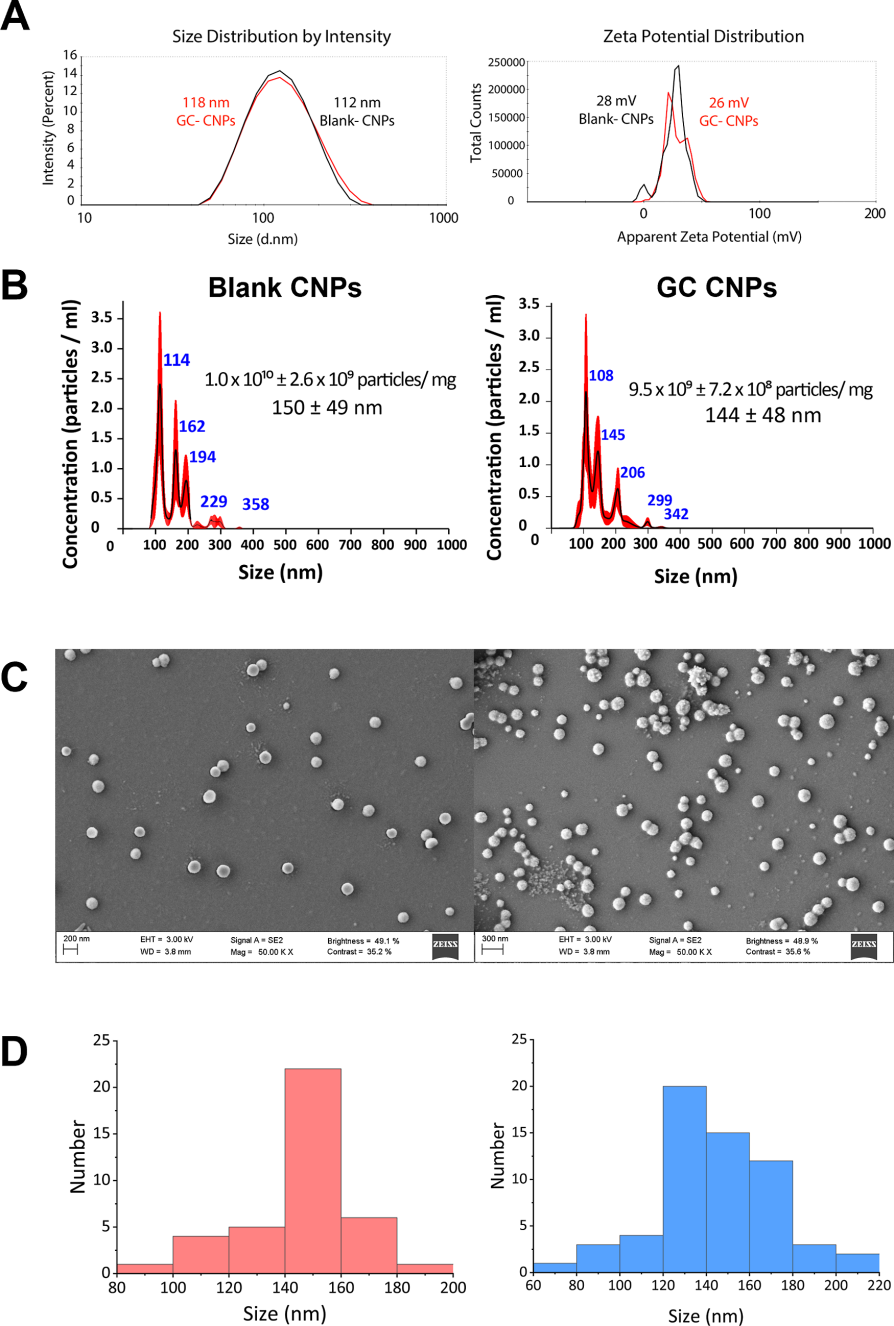
Particle size determination by Zetasizer, NP tracking analysis (NTA), and field emission scanning electron microscopy (FESEM) of the blank CNPs and GC-CNPS. (A) Particle size and zeta potential; (B) particle size distribution and the NP concentration of the blank and GC-CNPs, determined by NTA; (C) surface morphology of the blank CNPs and GC-CNPs determined by FESEM and shows that CNPs had a spherical shape; and (D) particle size distribution from the FESEM images calculated using ImageJ software.

DLS analysis of GC-CNP suspensions revealed the formation of NPs with an average diameter of 142 ± 18 nm and a zeta potential of +27 ± 3 mV. The PDI of the GC-CNPs was lower than 0.2, indicating the homogeneity of the NP population (Figure 2A). These values were very similar to those determined for blank CNPs, indicating an average diameter of 137 ± 21 nm and a zeta potential of +26 ± 2 mV.

NP tracking analysis (NTA) displayed that the blank CNPs and GC-CNPs had a similar average particle size of around 150 nm (Figure 2B), values close to those obtained previously by DLS. The results from scanning electron microscopy confirmed that the CNPs and GC-CNPs have narrow size distribution (Figure 2C). The SEM micrographs show that CNPs presented a near-spherical morphology and showed no signs of aggregation. The diameter of a hundred particles was measured from the micrographs of both the blank CNPs and GC-CNPs (Figure 2D). The average particle size was 147 ± 19 and 145 ± 28 nm for the blank CNP and GC-CNPs, respectively. No significant difference in the distribution pattern of the NPs was observed, even though the GC-CNPs had a slightly higher number of particles in the size range below 100 nm.

The encapsulation efficiency (EE) of GC in the CNPs was found to be 70 ± 3% that corresponds to 35 μg of protein per milligram of NPs, while the EE of PsaA used for comparison was <20%. When compared to PsaA, most of the amino groups of the lysine side chains were derivatized with acetyl thioacetate molecules or conjugated to Pn14TS. The higher encapsulation of GC into the NPs, when compared to PsaA, might be attributed to the diminution of surface-exposed positive charges. The prepared GC-CNPs contained approximately 6000 GCs per particle as calculated with particle numbers measured by NP tracking analysis (∼1.9 × 10^10^ particles per mg of GC-CNPs). The NPs freeze-dried in the absence of a cryoprotectant were redispersible in water but showed an increase in particle size. Both the blank and GC-CNPs freeze-dried in the presence of 5 and 10% trehalose as a cryoprotectant were easily re-dispersible after 1 week of storage at 4 °C. This facilitates the storage of nanovaccines in a dry powder form.

### *In Vitro* Evaluation of Dendritic Cell Viability in the Presence of CNPs

**Figure 3:**
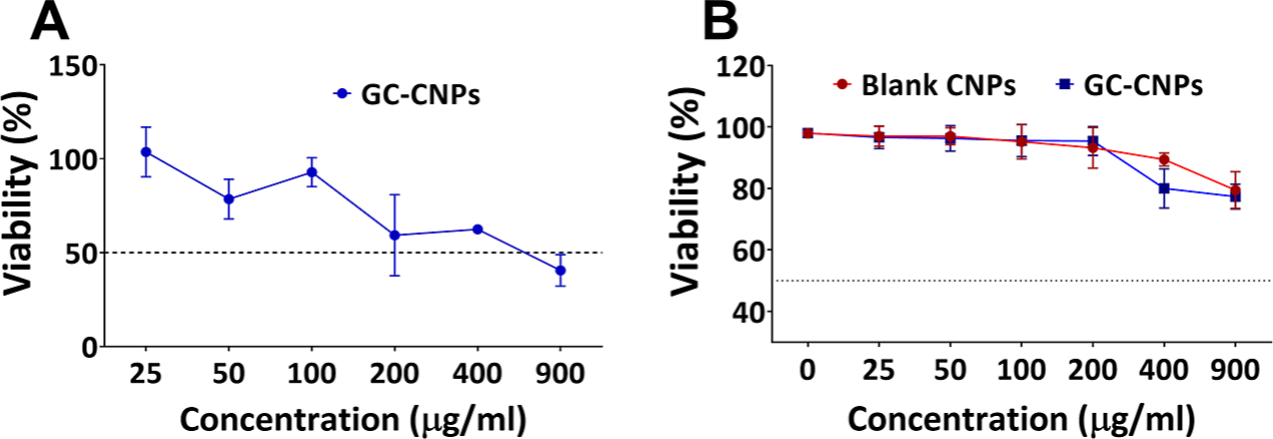
CNP cytotoxicity on iDCs. (A) MTS staining assay and (B) 7-AAD assay was performed for both GC-CNPs (blue lines) and blank CNPs (red lines). Results are presented as mean ± SD (*n* = 4). All the components used in the preparation of CNPs were assayed for the detection of endotoxin activity using the end-point chromogenic limulus amoebocyte assay (LAL test) (Supporting Information and Figure S2).

To study the influence of the GC-CNP concentration on the metabolic activity and/or survival of iDC, the MTS assay was performed to determine the cell survival in combination with metabolic activity. As can be seen in Figure 3A, over 80% of the immature dendritic cells (iDCs) were metabolically active when treated with GC-CNPs in the concentration range of 25–100 μg/mL, whereas the metabolic activity was reduced to 60% in the concentration range of 200–400 μg/mL and significantly reduced to 40% when treated with 900 μg/mL GC-CNPs. The results suggest that the GC-CNPs at a concentration of 25–100 μg/mL were in the acceptable nontoxic range. The toxicity seen at the higher concentrations might be due to the possibility of the NP aggregation at higher concentrations after 12 h. However, to mitigate the risk of the NP aggregation, the CNPs were used in the concentrations of 0.1 mg/mL or lower. In this regard, the GC-CNPs at a concentration of 50 μg/mL were adopted for further studies. In a likely manner, the cytocompatibility of the NPs with DCs was studied using the 7-AAD assay. The dose-dependent mortality of the DCs is observed in Figure 3B. The GC-CNPs at the concentration range of 25–200 μg/mL display over 90% survival and 80% at 400 μg/mL. At the highest concentration of 900 μg/mL, the DCs displayed 75% survival (see also Figure S1 for representative histograms). However, the mortality of the DCs was not more than 25% in any case. Both the blank CNPs and GC-CNPs had a similar profile of cytocompatibility and considered to be least toxic to DCs at concentrations below 200 μg/mL.

### GC-CNPs Are Effectively Internalized by iDCs

**Figure 4:**
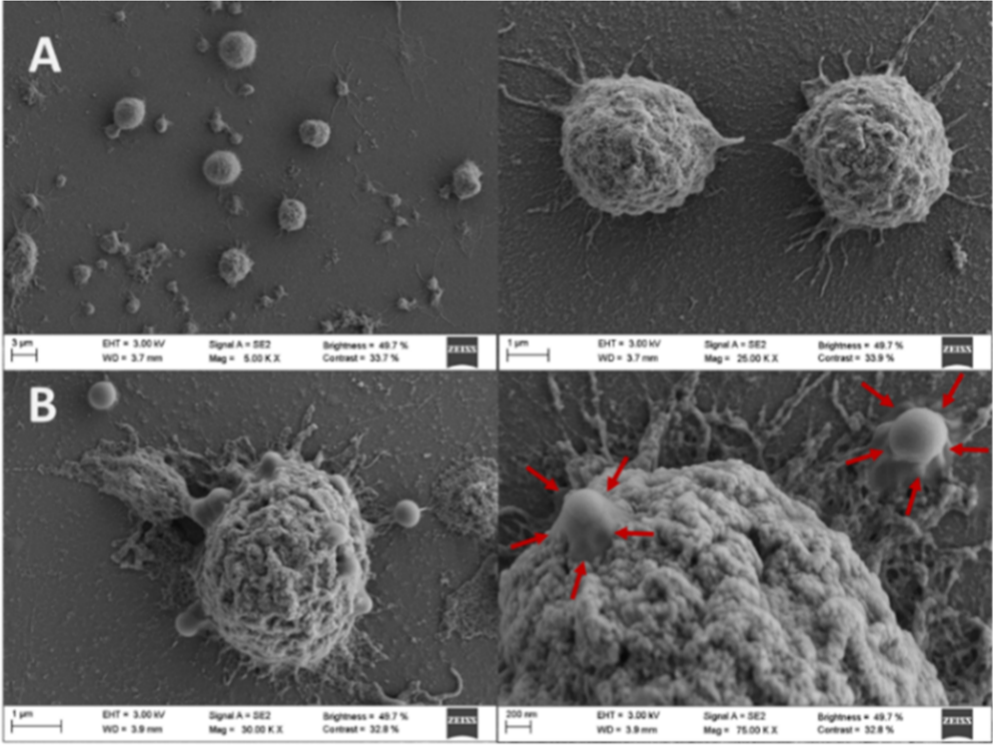
Assessment of GC-CNP uptake by monocytes using FEG-SEM. (A) Untreated iDCs and (B) iDCs treated with GC-CNPs. The GC-CNPs are pointed with the red arrow marks.

In Figure 4, from the micrographs obtained from FEG-SEM, the uptake of GC-CNPs by the iDCs can be observed upon 0.5 h of co-incubation. During the sample preparation for imaging, the iDCs undergo a series of washings to retain only the GC-CNPs firmly attached to the cell membrane, and this helps in the visualization of the NPs that are being internalized by the iDCs and not the NPs that are simply adsorbed to the cell surface. The red arrow marks in Figure 4B indicate the NPs that are being internalized by iDCs. This can be verified by comparing the captured image with the untreated iDCs shown in Figure 4A. Furthermore, the size of the internalized particles indicated in red arrows aligns with the size of the GC-CNPs, as determined by DLS and NTA.

**Figure 5:**
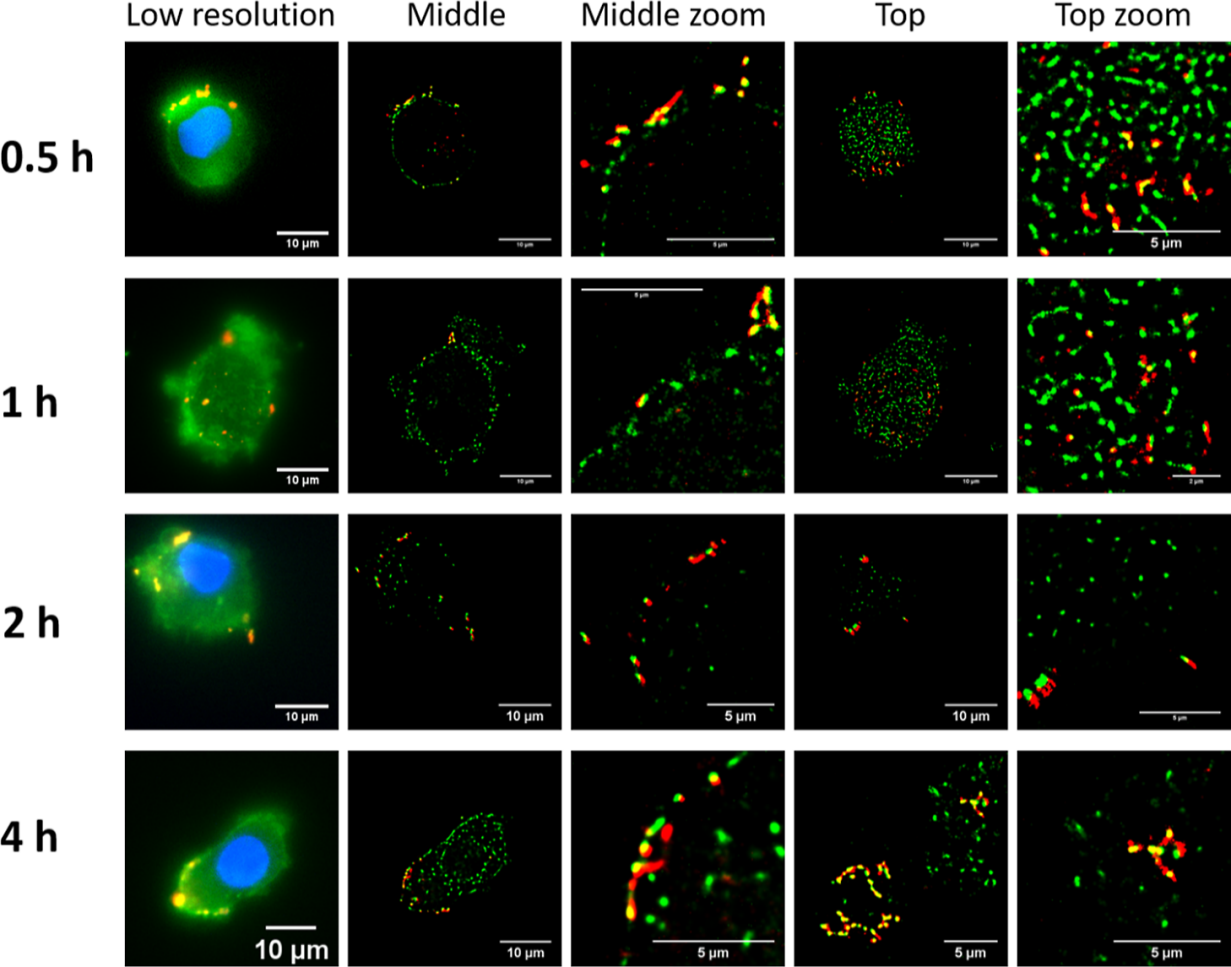
Internalization of Cy5-GC-CNPs (50 μg/million cells) by iDCs at different time points (0.5, 1, 2, and 4 h). The cell membrane is stained with wheat germ agglutinin-488 (WGA-488; green color), the nucleus stained with DAPI (blue color), and the NPs are labeled with Cy5 (red color).

**Figure 6:**
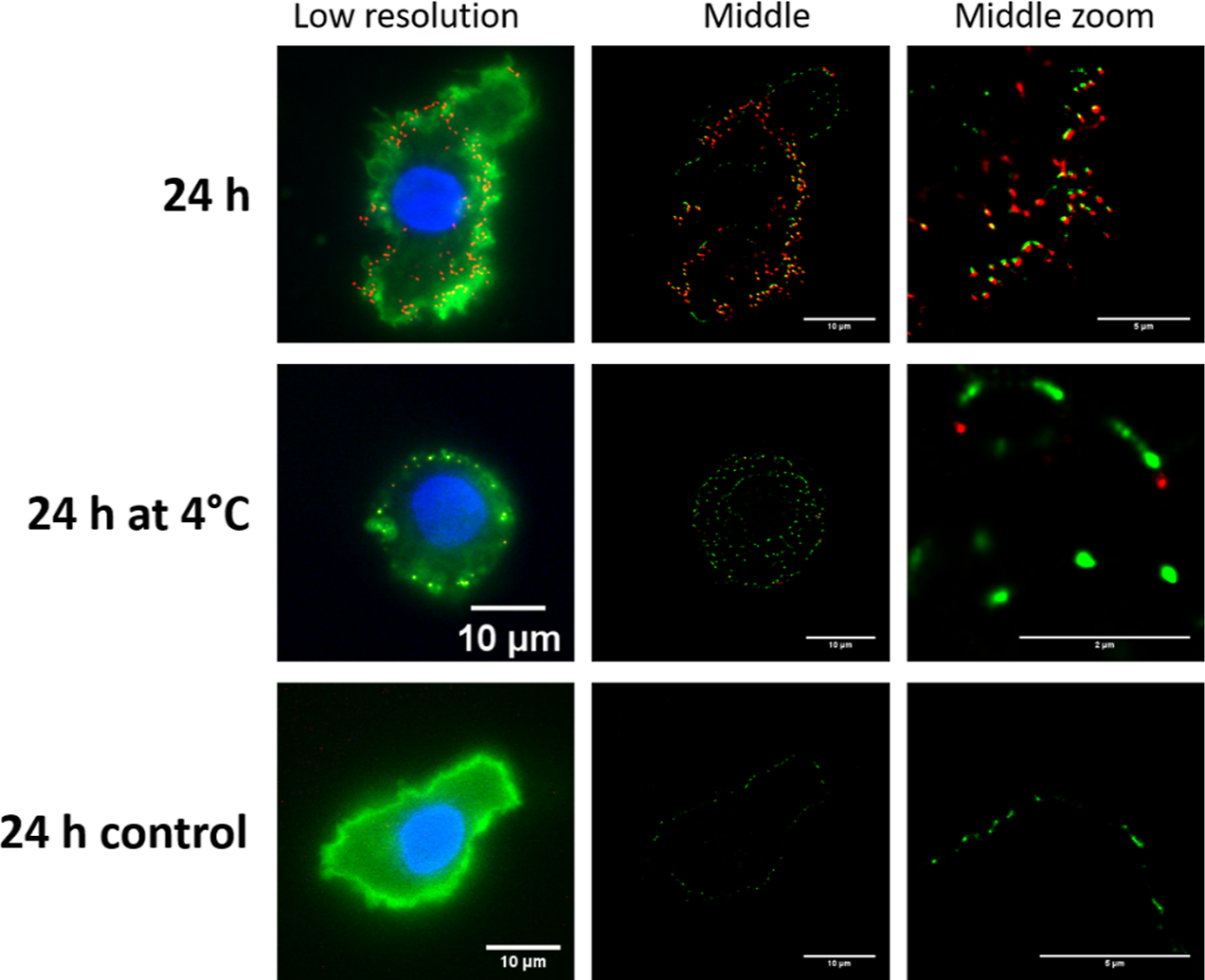
Internalization of the Cy5-GC-CNPs (50 μg/million cells) by MoDCs at 24 h (at 37 and 4 °C). The cell membrane is stained with wheat germ agglutinin-488 (WGA-488; green color), the nucleus stained with DAPI (blue color), and the NPs are labeled with Cy5 (red color).

Next the uptake of GC-CNPs in DCs was studied as the function of incubation time at different temperatures. The images in Figure 5 show that the internalization of the GC-CNPs increased with time. Each red dot in Figure 5 represents a NP that is present in the plane (0.5 μm thick) of the MoDC. Even though there is a gradual increase in the NP uptake with the time until 4 h, it was not maximum, and significantly higher NPs inside the cells were observed after 24 h (Figure 6). The images correspond to a cross-section of the MoDCs, measuring ∼0.5 μm in thickness. Consequently, the presence of NPs in these images is located in a limited region within the cell. The uptake of the CNPs was strictly temperature dependent. The NPs were rapidly internalized at 37 °C, while the DCs incubated at 4 °C displayed only a small number of NPs in the cells, and those were mostly present on the surface of the DCs. These results are in agreement with the studies on murine DC2.4 cell lines using super-resolution microscopy or DCs using flow cytometry and confocal microscopy (see the Supporting Information and Figures S3–S5).

### CNPs Enhance the Expression of the Co-stimulatory Molecules CD80 and CD86 on DCs

The study on the expression of co-stimulatory markers was carried out to elucidate the effect of GC-CNPs on DC maturation. The co-stimulatory marker CD86 is known to be a marker of primary DC maturation, while CD80 only increases in mature DCs. Many studies have shown that antigen delivery to DCs upregulates the expression of both CD80 and CD86 that are known to induce T-cell receptor signaling and promote T-cell activation.^44^ CD83 is most characteristic of cell surface markers for fully matured DCs, whose role is regulating the maturation of B and T lymphocytes.^45^

**Figure 7:**
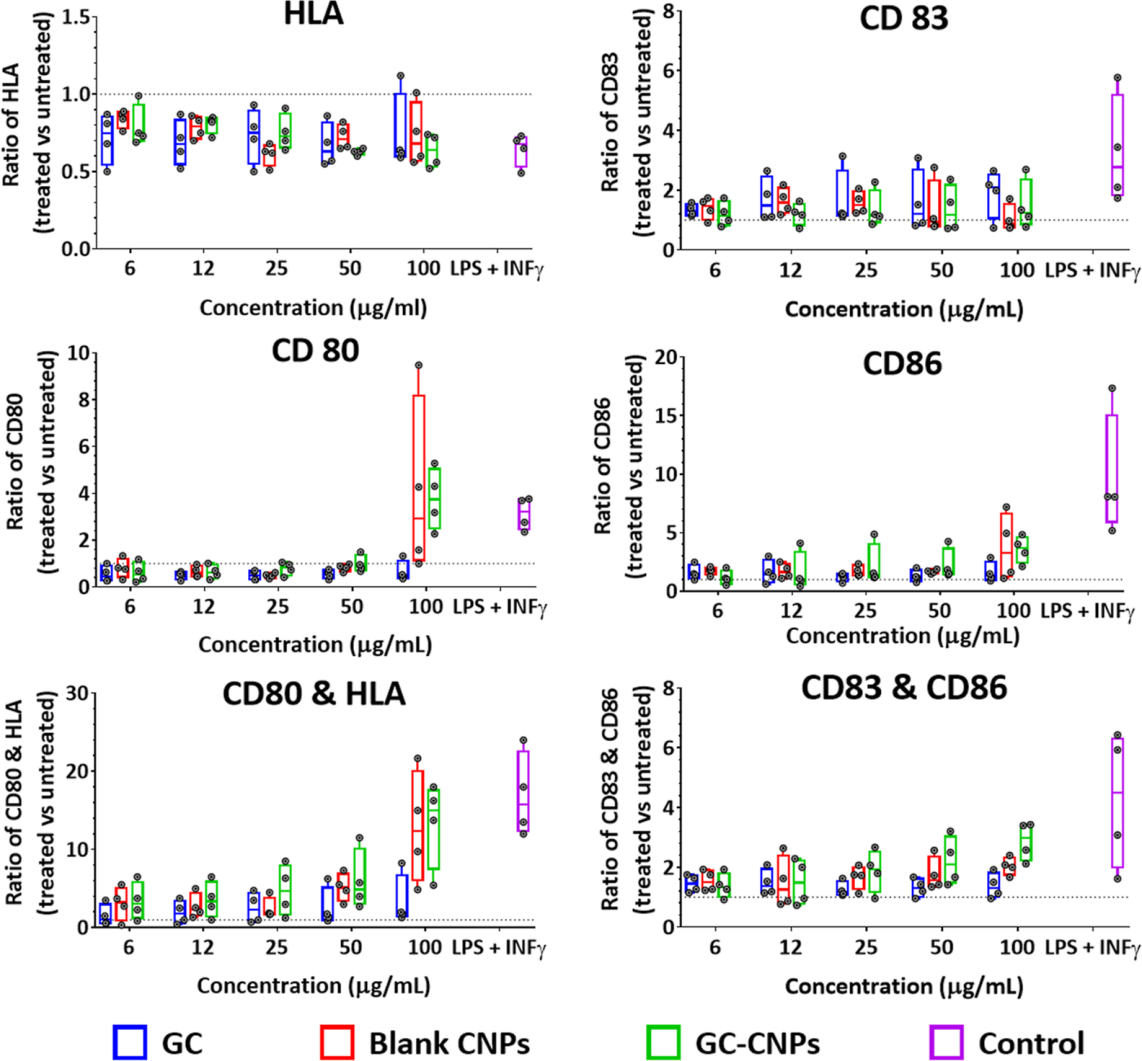
GC-CNPs induce iDC activation and maturation. The bars in the different colors indicate GC (blue), blank CNPs (red), GC-CNPs (green), and the control with LPS + INF-γ treatment (purple). The data represent the mean ± SD (*n* = 4).

As illustrated in Figure 7, iDCs treated with blank CNPs and GC-CNPs showed an enhanced expression of CD80, CD83, and CD86 markers, although this upregulation does not reach statistical significance, probably due to the natural inter-individual variability seen among the different donors. This phenomenon is frequently observed when using monocyte-derived dendritic cells and generally allows the indication of tendencies rather than statistically significant differences. Overall, there was no marked increase in the HLA expression with the same treatments, probably due to the high expression level already seen in untreated iDCs.

The stimulation of DCs with different concentrations of NPs showed that the upregulation and expression of CD86 occurred in a concentration-dependent manner and was highest at 100 μg/mL concentration. The CD80 upregulation was observed only at 100 μg/mL and not at the lower concentrations. The CD83 upregulation was observed when treated with NPs and was independent of their concentration. The DCs treated with blank CNPs and GC-CNPs displayed a similar profile of activation marker expression, but the upregulation was always higher in the case of GC-CNPs. This suggests that the presence of GC in the CNPs potentiate the stimulation of CD80, CD83, and CD86 by the DCs. The DCs treated with the GC alone showed similar CD83, lower CD86, and no CD80 upregulation when compared to the GC-CNP-treated DCs. Finally, a clear upregulation of all co-stimulatory molecules can be seen when iDCs were treated with LPS and INF-γ (Figure 7, violet columns) and higher for both CD83 and CD86 when compared to CNP-treated DCs but not for CD80.

These results can emphasize the role of CNPs as an adjuvant in DC stimulation. Chitosan is known for its role in macrophage activation and upregulation of the cytokines. There were low levels of endotoxin detected in our formulations (see Figure S1), and therefore, the obtained results are not due to endotoxin-derived activation. Overall, the results suggest that the NPs alone or in combination with GC can upregulate the co-stimulatory markers in comparison to the GC alone, indicating the adjuvant property of the CNPs.

### Effect of Nanoencapsulation on GC Immunogenicity

**Figure 8:**
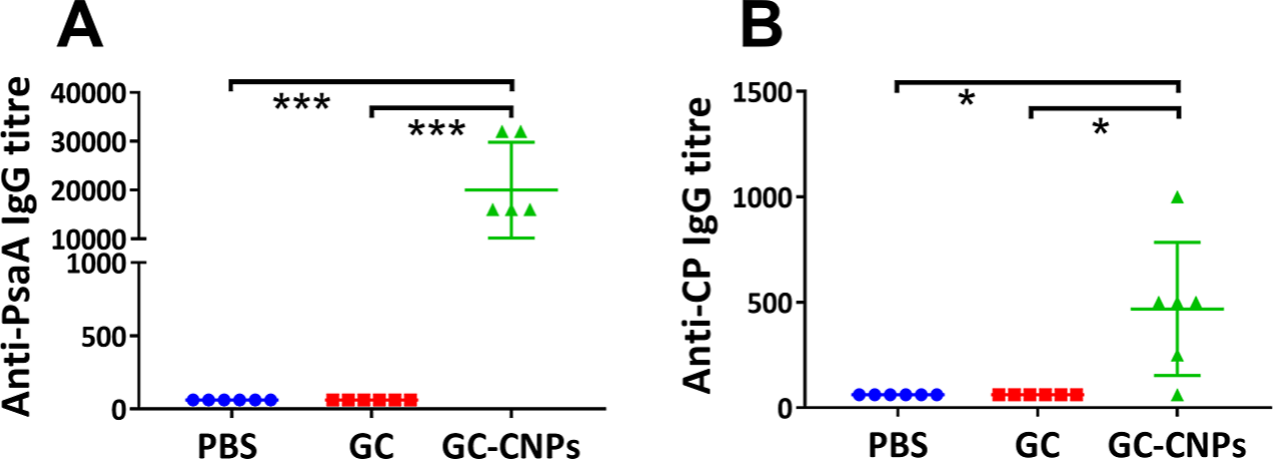
Antibody response in the mice immunized with PBS, GC, and GC-CNPs 2 weeks after the final immunization. The immunization was performed twice at days 0 and 14 and the serum antibody response in the mice was determined at day 21. The obtained results are represented as anti-PsaA IgG response (A) and anti-CP14 IgG response (B). Statistical difference between the groups is *P < 0.01, **P < 0.001, and ***P < 0.0005. Data represent mean ± SD (*n* = 6).

Groups of six mice were immunized twice at 2 week interval with GC, GC-CNP, or PBS adjuvanted with α-GalCer, and induced responses were analyzed by ELISA 1 week after the second immunization. The serum analysis shows no IgM response in any groups (data not shown). The secondary sera of mice immunized with blank CNPs do not show any anti-PsaA or anti-CP14 IgG response. Both anti-mPsaA and anti-CP14 Ab responses were low in the sera of mice immunized with GC, confirming our observations when using 3 μg of Pn14TS/dose.^13^ In contrast with these results, anti-mPsaA and anti-CP14 IgG responses were high when GC-CNPs were used as the immunogen. Anti-mPsaA and anti-CP14 were at least 100-fold higher and 10-fold higher, respectively, for the GC-CNP-treated mice in comparison to the GC-treated mice (*P* < 0.0005 and *P* < 0.05, respectively) (Figure 8).

**Figure 9:**
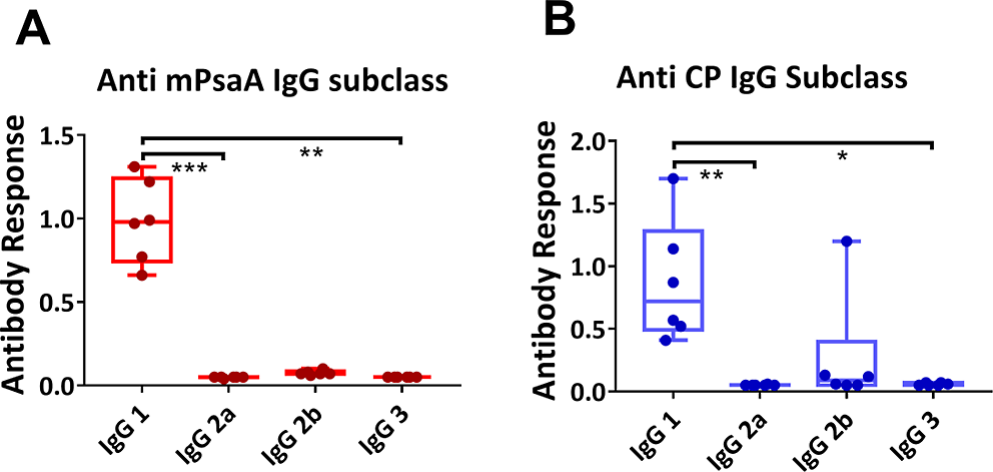
Subclass of anti-IgG antibody response in mice immunized with GC-CNPs 2 weeks after the final immunization. The immunization was performed twice at days 0 and 14, and the serum antibody response in the mice was determined at day 21. The obtained results are represented as anti-mPsaA IgG subclass response (A) and anti-CP IgG subclass response (B). Statistical difference between the groups is *P < 0.05, **P < 0.01, and ***P < 0.001. Data represent mean ± SD (*n* = 6).

In addition, further studies were performed to determine which subclass of IgG was predominantly expressed in the groups immunized with GC-CNPs. The serum samples of mice immunized with GC-CNPs displayed a high level of Abs of the IgG1 subclass and at a much lower extent of IgG2b against both PsaA and CP14 (Figure 9). There was no activation of other IgG subclasses, in particular of the IgG2a one. However, IgG2b was the second predominant IgG subclass that was in the mice immunized with GC-CNPs.

### *In Vivo* Assay of the Protective Effect of GC and GC-CNPs

**Figure 10:**
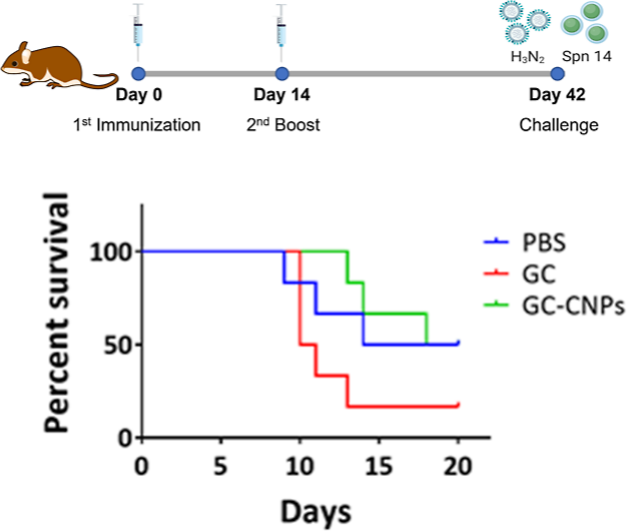
Survival of vaccinated mice. Mice were challenged IN by administering 30 cfu (colony forming units) of H_3_N_2_, followed by 1 × 10^6^ of *S. pneumoniae* serotype 14. The survival of the mice was monitored for 20 days. The differences between survival rates of six mice per group were analyzed by the Kaplan–Meier survival curve. Log-rank (Mantel–Cox) test *P* = 0.0714, no significant difference (*N* = 1).

Antibody response is important in the control of *S. pneumoniaein vivo*.^46^ To evaluate the efficacy of the GC-CNP formulation, we settled a model of pneumococcal infection in the mouse model. As *S. pneumoniae* serotype 14 is rapidly cleared in the mouse system, we embarked in a model of pneumococcal infection post-influenza. In this system, bacterial infection develops, and mice die from superinfection. Mice were immunized with GC, GC-CNPs, or PBS adjuvanted with α-GalCer. Treatment with α-GalCer has been shown to be associated with lower bacterial outgrowth in superinfected animals probably linked to an unspecific innate immune response stimulation.^47^ Mice were intranasally infected with a sub-lethal dose of IAV/Scotland/20/74 (H3N2) and challenged at 7 days post-influenza (dpi) with *S. pneumoniae* serotype 14. The invasive pneumococcal challenge (Figure 10) in the mice displayed 50% of survival on day 20 in the group immunized with SC or GC-CNPs, while the group immunized with an equivalent amount of GC showed only 17% survival. Notably, there was 100% survival until day 13 in the mice treated with GC-CNPs, whereas the survival reduced to 50% on day 10 in the groups treated with GC. Significant protection was also observed in the group of mice which received α-GalCer in PBS only. Overall, the results demonstrated that the mice immunized with GC-CNPs exhibited higher protection against the invasive pneumococcal challenge than GCs.

## Discussion

CNPs (CNPs) have earlier been investigated as a carrier for numerous vaccines,^37,48,49^ including pneumococcal protein vaccines,^31^ and have been tested as an adjuvant in a mixture with PCV13.^50^ To the best of our knowledge, chitosan or any other polymeric NPs have never been used as a carrier for the delivery of pneumococcal conjugate vaccines. The knowledge on the influence of the nanocarriers on the immunogenicity of the glycoconjugate is still missing.

CNPs have a proven track record in the delivery of therapeutic proteins and vaccines via a mucosal route.^51−53^ In addition, the CNPs have demonstrated to possess adjuvant activity in immunization and thus also appear as promising carriers for systemic administration.^54,55^ CNPs have the advantage of mild and solvent-free preparation that is ideal for preserving protein integrity during the encapsulation step. The incorporation of poloxamer 188 in the formulation enhances its stability^41^ and prevents the aggregation of the NPs during freeze-drying.^56^ The NPs freeze-dried in the presence of cryoprotectants did not show any increase in the hydrodynamic diameter of the NPs after re-dispersing. This helps in long-term storage of the NPs in the powder form. In addition, the presence of the cryoprotectant is known to protect protein conformation and structural integrity.^57^

The NPs used in our studies, both blank and antigen-loaded NPs, produced low levels of toxicity and were free from endotoxin contamination. The cell viability was more than 80% at concentrations below 100 μg/mL. This was confirmed by both MTS and 7 AAD assays. Performing both MTS and 7-AAD assays helps to know the number of cells that are metabolically active vs nonapoptotic. A similar study was performed by Das et al.^100^ to determine the survival of DCs in the presence of CNPs. They reported that the percentage DC survival when incubated with 100 μg/mL CNPs was ∼40% viability, while in our case, the viability was >80% at the same concentration. However, those CNPs had a size >250 nm and a zeta potential of +39 mV. It is possible that this higher zeta potential might have resulted in the increased toxicity.

The results obtained from super-resolution microscopy and flow cytometry suggest that the GC-CNPs are taken up through active pathways since the internalization does not take place at 4 °C. The imaging shows that the GC-CNPs are progressively taken up to localizing in the cytoplasm. As a result of the uptake, we found that both blank CNPs and GC-CNPs but not GC alone were able to promote the expression of costimulatory markers like CD80, CD83, and CD86. Contrasting with these results, Han et al. reported an upregulation of costimulatory markers like CD80, MHC II, and CD86 only for the antigen-loaded CNPs in comparison to the antigen alone or blank CNPs.^58^ However, Franco-Molina et al. demonstrated CD80 and CD83 upregulation with blank CNPs and the differentiation of human monocytes into iDCs.^59^ Thiele et al. reported that upregulation of CD83 is associated with the NP uptake by phagocytosis, which supports our results that show GC-CNP uptake by phagocytosis.^60^

The present study aimed to evaluate the immunogenicity of the pneumococcal glycoconjugate, whether naked or encapsulated, when SC was administered in mice. GC was poorly immunogenic in mice when administered SC in comparison with our previous experiments (Figure 8).^13^ However, in the present study, GC was administered at a 6 time lower dose. The groups immunized with GC-CNPs displayed 10- and 100-folds greater anti-CP14 and anti-PsaA IgG response, respectively, in comparison to GC (Figure 8). Strikingly, the IgG response induced by the GC-CNPs in mice was also higher than that previously observed for GC administered at a 6 time higher dose.^13^ This is in agreement with previously published results where the encapsulation of the antigen in the CNPs led to a 10-fold antigen dose reduction without affecting the level of the immune response.^61,62^ The IgG antibodies generated by GC-CNPs were predominantly IgG1 subclass. In the literature, it can be seen that the IgG1 subclass of antibodies is mainly induced by bacterial proteins, while the IgG2 antibodies are induced by capsular polysaccharides.^63^ The results obtained from our studies showed greater IgG1 production against both PsaA and CP, while no IgG2a or IgG3 response was observed. Similar results were observed by Bal et al. when antigen-loaded trimethyl CNPs were injected intradermally.^55^ The mice treated with GC-CNPs also displayed CP14-specific IgG1 response; this is classically ascribed to the conjugation of the carbohydrate hapten, herein Pn14TS, to the carrier protein, herein mPsaA. Interestingly, IgG2b against CP14 was the second prevalent subtype. The results are in agreement with the studies performed by González-Miro et al., where the mice immunized with self-assembled PsaA particles produced predominantly IgG1 and IgG2b as a second predominant response.^64^ The generation of anti-IgG1 Abs against both PsaA and CP can be attributed to the activation of the phagocytic response.^64^ The generation of IgG1 subclass antibodies corresponds to the Th2 (humoral) immune response, and the IgG2b subclass is identified as a part of Th1 (cell-mediated) immune response.^64,65^ The results are in agreement with the hypothesis that a vaccine based on PsaA can elicit both humoral and cellular immune response.^66^ Next, the functionality of Abs induced in mice was assessed using an IAV infection challenge. The mice immunized with GC-CNPs displayed greater protection over its counterparts immunized with GC.

## Conclusions

In this study, we reported CNPs as a potential carrier for a semisynthetic glycoconjugate antigen. The NPs were rationally designed to encapsulate, control the release of the antigen, and maintain the stability in biological media. Moreover, the NPs could be easily freeze-dried and reconstituted, indicating the possibility for the development of a thermostable dry powder formulation. NPs were highly internalized by the dendritic cells, and the uptake was increasing with the time. Encapsulation of GC in the CNPs enhanced the expression of co-stimulatory markers when compared to the naked GC or blank CNPs. Overall, this study revealed that CNPs can enhance the immunological properties of both pneumococcal protein and carbohydrate components of the glycoconjugate vaccine, encouraging the further study of the formulation as nanoparticulate vaccine delivery systems for preventing the infections against *S. pneumoniae*.

## Experimental Section

### Reagents

Chitosan, poloxamer 188, and sodium tripolyphosphate were purchased from HMC^+^, Sigma-Aldrich, and BSAF, respectively. The cytokines IL-4, GM-CSF, and INF-γ were procured from Miltenyi Biotech. PBS (pH 7.2), RPMI 1640, PSG 100×, and fetal bovine serum (FBS) were obtained from Gibco, Life Technologies. Ficoll Histopaque 1077, paraformaldehyde, DAPI, Triton X-100, and pentasodium tripolyphosphate were procured from Sigma-Aldrich. All the antibodies used for FACS analysis were obtained from either Miltenyi Biotech or Sigma-Aldrich. The Pierce Chromogenic Endotoxin Quant Kit (A39552) was procured from Thermo Fisher Scientific. Cy5-NHS was purchased from Lumiprobe. The gel filtration columns (Centripure PD10 columns) were obtained from EMP Biotech. PsaA and GC were produced in US2B at Nantes Université.^14^ All the other chemicals and reagents used were of analytical grade.

### Preparation and Characterization of CNPs

First, 0.5 mL of chitosan solution (2 mg/mL) and 0.5 mL of poloxamer solution (20 mg/mL) in ultrapure H_2_O were mixed under magnetic stirring. Next, 0.5 mL of TPP solution (0.4 mg/mL) in ultrapure H_2_O was added at once to the mixture of chitosan and poloxamer under magnetic stirring (700 rpm). After 30 min of the reaction, the NPs were concentrated by centrifugation at 12,000 RCF, for 12 min, at 15 °C, using 10 μL of glycerol bed. After the centrifugation, the pellet in the bottom was carefully collected and resuspended in ultrapure water. For GC-loaded CNPs, the TPP solution (0.5 mL of 0.4 mg/mL) containing GC (50 μg) was added to 1 mL of 0.1% w/v chitosan in 1% poloxamer kept under magnetic stirring at 700 rpm.

The particle size and polydispersity index of the NPs were measured by dynamic light scattering (DLS), and the zeta potential was calculated from the electrophoretic mobility values obtained by laser Doppler anemometry using a Zetasizer Nano-ZS90 (Malvern Instruments; Malvern, UK). All the measurements were performed at 25 °C with a detection angle of 173° in distilled water unless otherwise indicated. The NP concentration and stability in cell culture medium was evaluated by NP tracking analysis using NanoSight NS500 (Malvern Instruments; Malvern, UK). The surface morphology of the GC-CNPs was examined by field emission scanning electron microscopy (FESEM, ZEISS FESEM ULTRA Plus, Germany). For FESEM studies, 10 μL of the GC-CNP suspension (10,000 times diluted) was placed on the silicon wafer and left to dry overnight in the desiccator. Prior to the analysis, the samples were sputter-coated with iridium (10 nm thickness). For the morphological analysis by STEM, 10 μL of the GC-CNP suspension (10,000 times diluted) was deposited on the copper grid, stained with 2% phosphotungstic acid, and washed with ultrapure water, and the grids were left to dry overnight in the desiccator. The blank and GC-CNPs (0.5 and 1% w/v) were lyophilized in the presence of 5 and 10% (w/v) trehalose as a cryoprotectant. The samples were frozen overnight at −20 °C and then transferred to the freeze-dryer Genesis 25 ES, VirTis Model-Wizard 2.0 (SP Industries, USA). The primary drying step lasted for 35 h during which the temperature was gradually increased from −40 to −20 °C. This was followed by a secondary drying step in which the temperature was increased to RT from 0 °C. After the freeze-drying, the nanoparticles were resuspended in ultrapure water and analyzed for their particle size and PDI. A similar freeze-drying process was used to determine the formulation process yield.

### Encapsulation of GC into CNPs and Release Studies

The EE of GC in CNPs was determined by calculating the amount of free GC present in the collected supernatant after the centrifugation. The amount of free GC was determined using the micro-BCA assay kit (Thermo Fisher Scientific) by measuring the absorbance at 562 nm. The quantification was performed using the linear standard curve produced with the solutions of GC solubilized in the supernatant of CNPs in the concentration range of 0.5–10 μg/mL. The % EE is calculated as follows: % EE = *A* – *B*/*A* × 100, where *A* is the total GC and *B* is the free GC in the supernatant.

### Fluorescence Labeling of Chitosan

The Cy5-labeled GC-CNPs were prepared to visualize the GC-CNPs during cell studies. Briefly, chitosan (50 mg; 0.3 mmol monomer units; 1 equiv) was placed in a reaction vial and dissolved in deionized water (5 mL). Cyanine (Cy5) (1 mg; 0.015 mmol; 0.050 equiv) in dimethyl sulfoxide (DMSO) (0.3 mL) was added to the chitosan solution under continuous stirring. After sealing, the vial was stirred at RT for 16 h and protected from light. After the reaction, the labeled chitosan was purified using PD10 columns (Centri Pure P10; Zetadex Gel Filtration columns). The yield of the reaction was measured afterward by weighing the freeze-dried product in the Eppendorf and subtracting the weights. The labeling of the GC-CNPs was simply performed by substituting 2% of chitosan with the Cy5-labeled chitosan (Cy5-chitosan) during the synthesis of the CNPs. Particle size analysis shows that the Cy5-GC-CNPs had an average diameter of 150 ± 9 nm and a zeta potential of 30 ± 2 mV. The formulation contained approximately ∼8–15 thousand Cy5 molecules per particle as calculated from particle numbers obtained by NTA.

### Donor Selection and Blood Collection

Buffy coats used in the experiments were obtained from anonymous donors and were kindly donated by the Agency for the Donation of Organs and Blood (ADOS, Santiago de Compostela) with the approval of the Director of the Agency and the Clinical Research Ethics Committee of Galicia (2014/543). Freshly obtained buffy coats were used within 24 h. Heparinized blood samples were obtained from healthy volunteers among the staff of Faculty of Pharmacy, University of Santiago de Compostela, and CiMUS. Before the collection, informed consent was obtained from the volunteers in accordance with the guidelines of the Ethical Committee of Clinical Research of Galicia.

### DC Generation

Peripheral blood mononuclear cells (PBMCs) were isolated by the Ficoll gradient centrifugation method. Adherent monocytes were isolated by incubating PBMCs in culture plates (2 h, 37 °C) in R2 culture media (RPMI-1640 completed with 2% of FBS). After the incubation period, nonadherent cells were washed thrice with PBS, and adherent monocytes were cultured for 6 days in R10 media (RPMI-1640 completed with 10% FBS) supplemented with GM-CSF and IL-4 (100 ng/mL each). After 3 days, half of the media was replaced with fresh R10 supplemented with equal amounts of cytokines. The resultant immature DCs (iDCs) were scraped and collected from the culture plates on day 6. These iDCs were used for further experimentation. Mature DCs (mDCs) were obtained by incubation of iDCs with bacterial lipopolysaccharide (LPS) (10 ng/mL) and interferon-γ (IFN-γ) (100 U/mL) for 48 h. Murine DC2.4 cells were cultured in RPMI 1640 medium, which was supplemented with 10% fetal bovine serum and penicillin/streptomycin antibiotics.

### Cytocompatibility Studies

The cytocompatibility assay of GC-CNPs in the DCs was performed by the MTS assay. The generated iDCs (1 × 10^5^ cells/mL) were incubated with different concentrations of the CNPs (25, 50, 100, 200, 400, and 900 μg/mL) for 24 h. The untreated iDCs were used as a positive control. After 24 h of incubation with the CNPs, the cells were washed with PBS and were incubated with fresh R10 media for another period of 24 h, and 20 μL of MTS reagent was added to each well after 20 h and incubated for another 4 h. The intensity of the color was quantified by measuring the absorbance at 490 nm using a spectrophotometer. The results were plotted as percentage (%) viability vs the NP concentration. The percentage viability is a difference of metabolically active cells in the untreated group and the groups treated with the NPs.

The cytocompatibility of blank CNPs and GC-CNPs was also studied using 7-amino actinomycin D (7-AAD). Briefly, following the incubation of iDCs with the blank CNPs or GC-CNPs (25, 50, 100, 200, 400, and 900 μg/mL) for 24 h, cells were harvested, washed twice with PBS, and stained with 7-AAD 1 μL per tube (0.05 μg/μL) for 30 min at 4 °C. After washing three times with PBS containing 2% BSA, the cells were analyzed by flow cytometry (BD FACSCalibur cytometer). Data were analyzed using Flowing software (Cell Imaging Core, Turku Centre for Biotechnology, Finland). Data are shown as the percentage of cells viable after incubating with the different concentrations of NPs.

### Internalization of NPs by DCs

The surface morphology of the macrophages incubated with the NPs (50 μg/mL, for 0.5 h, at 37 °C) was performed using FESEM (ZEISS FESEM ULTRA Plus, Germany).

To track the NPs after internalization by dendritic cells, the NPs and the dendritic cells were labeled with two different dyes. The NPs labeled with Cy5. DCs (5 × 10^5^ per well) were plated into a 24-well plate with 0.5 mL of R10 media. Following that, the cells were incubated with the Cy5-labeled GC-CNPs (50 μg/million cells) for different time periods (0.5, 1, 2, 4, and 24 h). After the incubation time, the DCs were washed with PBS. Afterward, the cells were fixed with 4% PFA for 15 min. To understand the position of the NPs inside cells, the plasma membrane of the MoDCs was stained with Alexa 488-labeled WGA at 0.2 μg/mL (wheat germ agglutinin, a lectin known to bind to *N*-acetyl-d-glucosamine and sialic acid on the cell membrane), and the nucleus was stained using DAPI. The staining was performed in Lab-Tek 8 well plates for 10 min, and the DCs were washed twice with PBS after incubation. The cells were suspended in 20–30 μL of STORM buffer (160 μL of PBS + 20 μL of 50% glucose + 20 μL of β-mercaptoethanol + 2 μL of glucose oxidase) just before imaging.

### Study on the Effect of GC-CNPs on DC Maturation by Phenotypic Analysis

For the phenotypic analysis, the iDCs were seeded on 24-well plates at a density of 5 × 10^5^ cells per well 0.5 mL of R10 media. The DCs were incubated for 48 h at 37 °C in R10 with blank or GC-CNPs using LPS (10 ng/mL) as a positive control. After 48 h of incubation, the generated DCs were collected and washed twice (300*g*, 7 min, 4 °C) with PBS containing 0.1% BSA. DCs were resuspended in 200 μL of PBS with 1% FCS (1% PBS); of this, 100 μL of the DCs was incubated with 50× diluted anti-CD83-PE, anti-CD86-APC, and anti-CD1a, another 100 μL with 50× diluted anti-CD80-PE, anti-HLA-APC, and anti-CD1a-FITC (in all cases, for 30 min, in the dark, on ice). CD1a was included as a DC marker to verify correct monocyte differentiation. After the 30 min staining period, the cells were harvested, washed thrice with 1% PBS, and resuspended in 500 μL of PBS. The DCs were quantified for the expression of CD80, CD83, CD86, and HLA using flow cytometry (BD FACSCalibur flow cytometer; BD Biosciences, Madrid). The cytometry data were analyzed using the Flowing software program (Cell Imaging Core, Turku Centre for Biotechnology, Finland). The forward versus side scattering was used for gating the live cells, and the CD1a-positive cells were picked for the quantification of co-stimulatory markers. The expression of cytokines from the CNPs-treated DCs were compared against the LPS-treated DCs, assuming 100% maturation.

### Immunization Studies in Mice

Specific pathogen-free C57BL/6 mice (6 weeks-old, female) were purchased from Janvier (Le Genest-St-Isle, France). Mice were maintained in a biosafety level 2 facility in the Animal Resource Centre at the Lille Pasteur Institute for at least 2 weeks prior to usage to allow appropriate acclimation. Mice were fed a standard rodent chow (SAFE A04) (SAFE, Augy, France) and water ad libitum. The diet contains ∼11.8% fiber including ∼10% water-insoluble fiber (3.6% cellulose) and 1.8% water-soluble fiber. All the animal experiments were performed at Lille Pasteur Institute according to the ethical guidelines (agreement number AF 16/20090 and 00357.03).

Groups of six female C57BL/6 mice were injected SC with GC or GC-CNPs (0.6 μg of carbohydrate antigen/5 μg protein/dose). All mice were administered with 250 ng of α-GalCer as an adjuvant. Three groups of mice were immunized with SC on day 0 and boosted at day 14 with PBS, GC, or GC-CNP. Mice were bled 1 day prior the first immunization and 1 week after every immunization. Sera were stored at −80 °C until the quantification of Ab response by ELISA.

### Measurement of Humoral Response

The Ab responses induced upon immunization were assessed by using enzyme-linked immunosorbent assay (ELISA) as described previously, with slight modifications. In brief, the samples with different serial dilutions were loaded into individual 96-well microtiter Nunc Maxisorp (Thermo Fisher Scientific), where the plates were coated with mPsaA (0.1 μg/well) or Pn14TS (0.2 μg/well) and plates overnight at 4 °C. The goat anti-mouse IgA, IgM, and IgG(H + L)-HRP-labeled conjugate (CliniSciences) used as secondary Ab at a dilution of 1/6000 was used as secondary antibodies. The reactions were read in an Infinite M1000 spectrophotometer (TECAN). Similarly, anti-mouse IgG1, IgG2a, IgG2b, and IgG3 were used to determine the predominant IgG subclass expressed. To determine the anti-CP14 response, the purified capsular polysaccharide (CP14) (Alliance Bio Expertise, France) was coated in the wells. The Ab titer was defined as the dilution of immune serum that gave an OD (405 nm) at least twice that observed with pre-immune serum.

### Influenza A Virus Infection

The *S. pneumoniae* (serotype 14) strain used in this study was a gift from Dr M. de Jonge (Nijmegen University, The Netherlands). The mouse-adapted H3N2 IAV strain Scotland/20/1974 was described in ref (67). For infection with IAV, 50 μL of phosphate-buffered saline containing (or not, in a mock sample) 30 plaque-forming units (PFU) of the H3N2 IAV strain Scotland/20/1974 was intranasally administered to anesthetized mice. This dose corresponds to a sub-lethal dose, which is necessary to investigate secondary bacterial infection. For secondary pneumococcal infection post-influenza, mice were challenged (I.N) at 7 dpi with *S. pneumoniae* (1 × 10^6^ PFU). The survival of the mice was monitored for 20 days.^68^

### Statistical Analysis

The two-way ANOVA Bonferroni’s multiple comparison test, Mann–Whitney *U* test, one-way ANOVA with Kruskal–Wallis analysis by Dunn’s multiple comparison ,test and one-way ANOVA with Bonferroni’s multiple comparison test were used to calculate the statistical significance. A probability value of *P* < 0.05 was considered statistically significant.

## Supporting Information Available

Representative histograms; endotoxin analysis; internalization of Cy5-GC-CNPs by DC2.4 cell lines, MoDCs, and DCs; and associated experimental part (PDF)

## Abbreviations

α-GalCer: α-galactosyl ceramide
7-AAD: 7-aminoactinomycin D
APC: allophycocyanin
DCs: dendritic cells
DLS: dynamic light scattering
DAPI: 4′,6-diamidino-2-phenylindole
FESEM: field emission scanning electron microscopy
GM-CSF: granulocyte-macrophage colony-stimulating factor
iDCs: immature dendritic cells
I.N: intranasal
LAL: limulus amoebocyte lysate
LPS: lipopolysaccharide
MoDCs: human monocyte-derived dendritic cells
MTS: (3-(4,5-dimethylthiazol-2-yl)-5-(3-carboxymethoxyphenyl)-2-(4-sulfophenyl)-2*H*-tetrazolium)
NTA: nanoparticle tracking analysis
PCV: pneumococcal conjugate vaccine
PPSV: pneumococcal polysaccharide vaccines
S.C: subcutaneous
STORM: stochastic optical reconstruction microscopy
TPP: tripolyphosphate
WGA: wheat germ agglutinin
