# Supplementary material for: Semisynthetic Pneumococcal Glycoconjugate Nanovaccine
Source: Bioconjug Chem. 2023 Sep 11;34(9):1563–75. doi: 10.1021/acs.bioconjchem.3c00252 (PMC10515484; doi:10.1021/acs.bioconjchem.3c00252)
Supplement: Supplementary file 1 — bc3c00252_si_001.pdf [file bc3c00252_si_001.pdf]

A Semisynthetic Pneumococcal Glycoconjugate Nanovaccine

Maruthi Prasanna[†, ‡, §, ↟], Rubén Varela Calvino[§], Annie Lambert[‡], Maria Arista Romero[°], Sylvia Pujals[°], François Trottein[#], Emilie Camberlein[‡], Cyrille Grandjean[*, ‡], Noemi Csaba[*, †].

[†] Center for Research in Molecular Medicine and Chronic Diseases, Department of Pharmacology, Pharmacy and Pharmaceutical Technology, University of Santiago de Compostela, Spain

[‡] Nantes Université, CNRS, Unité des Sciences Biologiques et des Biotechnologies (US2B), UMR 6286, F-44000 Nantes, France

[§] Department of Biochemistry and Molecular Biology, University of Santiago de Compostela, Spain

Univ. Lille, CNRS, INSERM, CHU Lille, Institut Pasteur de Lille, U1019 - UMR 9017 - CIIL - Center for Infection and Immunity of Lille, F-59000 Lille, France

° Department of Biological Chemistry, Institute for Advanced Chemistry of Catalonia (IQAC-CSIC), 08034 Barcelona, Spain

TABLE OF CONTENTS

Figure S1 S2

Endotoxin testing S2

Figure S2 S3

Internalization of GC-CNPs in DC2.4 cell lines after different time periods of incubation S3

Figure S3 S4

The study on internalization of nanoparticles by DCs by cytometry and laser confocal scanning microscopy (LCSM) S4

Figure S4 S5

Figure S5 S7

References S7

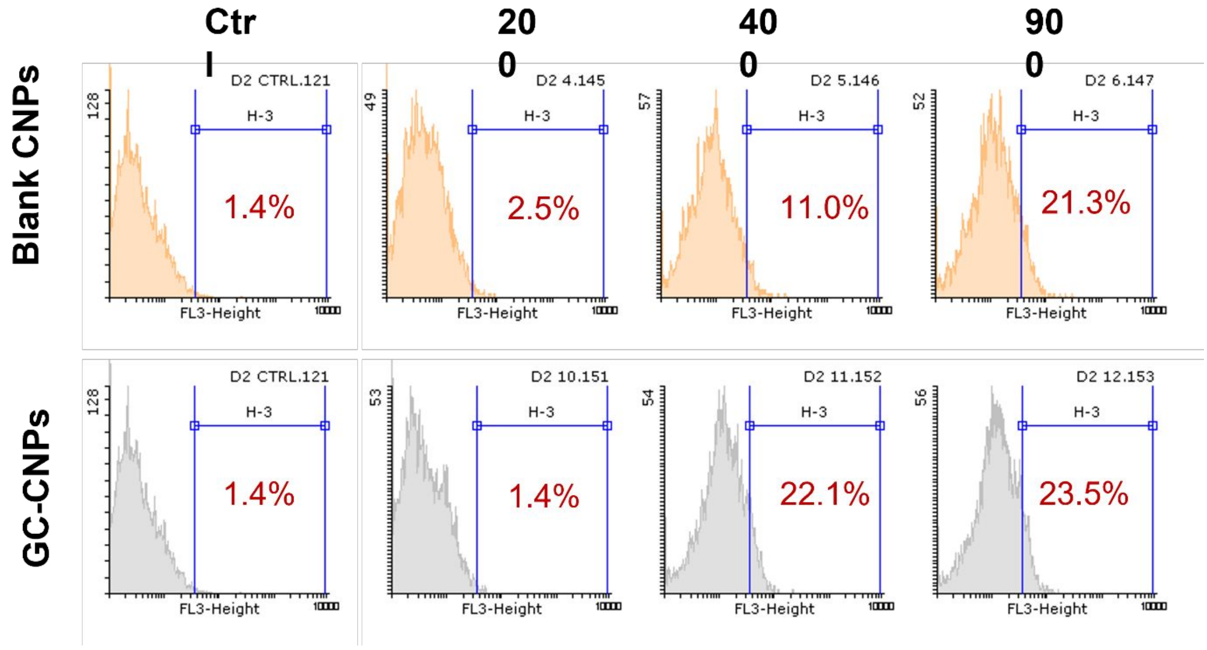

Figure S1. Representative histograms obtained from flow cytometric analysis after 7-AAD staining of GC-CNPs (Top) and blank CNPs (Bottom). Results are presented as mean± SD from 4 donors.

Endotoxin testing

The quantification of endotoxins in the samples used for DC cell studies was performed using the Pierce Chromogenic Endotoxin Quant Kit (A39552). Briefly, 2 micrograms of antigen (PsaA or GC) or 50 micrograms of chemicals (Chitosan, Kolliphor-188 or TPP) were suspended in water and tested for the presence of endotoxin according to the manufacturer's instructions.[1] The calibration curve (0.1–8 EU/mL) was constructed using a stock solution of 10 EU/mL. To avoid external endotoxin interference, all the dilutions were prepared in endotoxin-free water, which was also used as blank. All the tubes and tips used to perform this quantification were endotoxin-free and the entire procedure was performed inside of a laminar flow cabinet. Endotoxin content was lower than 0.5 EU/µg in all the cases (**Figure S2**), which are far below the maximum recommended endotoxin levels for recombinant subunit vaccines (20 EU/mL).[2] The colloidal stability of the nanoparticles was evaluated by measuring their size and PDI in the R10 medium and was found to be concentration dependent. The aggregation of the CNPs was observed when the concentrations were above 500 µg/ml in R10 media after 24 h. Below this concentration, the NPs showed no signs of aggregation.

A

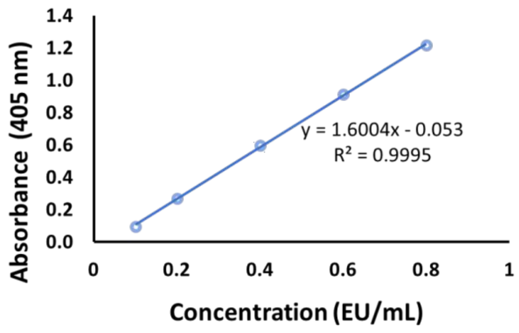

B

Formulation Components	ng/µg	EU/ µg
mPsaA	0.000734	0.007342
GC	0.035147	0.351475
Chitosan	0.000004	0.000037
Poloxamer	0.000009	0.000095
TPP	0.000003	0.000033

Figure S2. Endotoxin analysis of CNPs formulation components. A) The calibration curve used to quantify the endotoxin level in the formulation components. **B)** Endotoxin quantification in the different CNPs components. As can be seen, all of them shows levels below 0.5 EU/µg.

Internalization of GC-CNPs in DC2.4 cell lines after different time periods of incubation

The DC2.4 cells (25,000 per well) were seeded on Lab-Tek plates with 0.2 mL of R10 media. Following that cells were incubated with the Cy5 labelled GC-CNPs (0.5 µg of NPs) for different time periods (0.5, 1, 2, 4 and 24h). After the incubation time, the DCs were washed with PBS. Afterwards, the cells were fixed with 4% PFA for 15 min. For imaging, the cells were stained using DAPI and wheat germ agglutinin (WGA, at 0.2 µg/ml) for 10 min, and the DCs were washed twice with PBS after incubation. The cells were suspended in 20-30 µl of STORM buffer.

Figure S3 clearly displays that NPs internalization increases with time. With the increase in time, the NPs concentration increased both on the surface and the centre of the cells, which indicates the NPs were continuously internalized throughout the incubation time and not just during the initial hours of incubation. The images from the mid-section of the cells show the NPs concentration in the cytoplasm increases with the time, and the NPs were present around the nucleus and were not observed inside. The results obtained from these studies are identical to that of nanoparticle uptake by MoDCs (Manuscript, Figure 6).

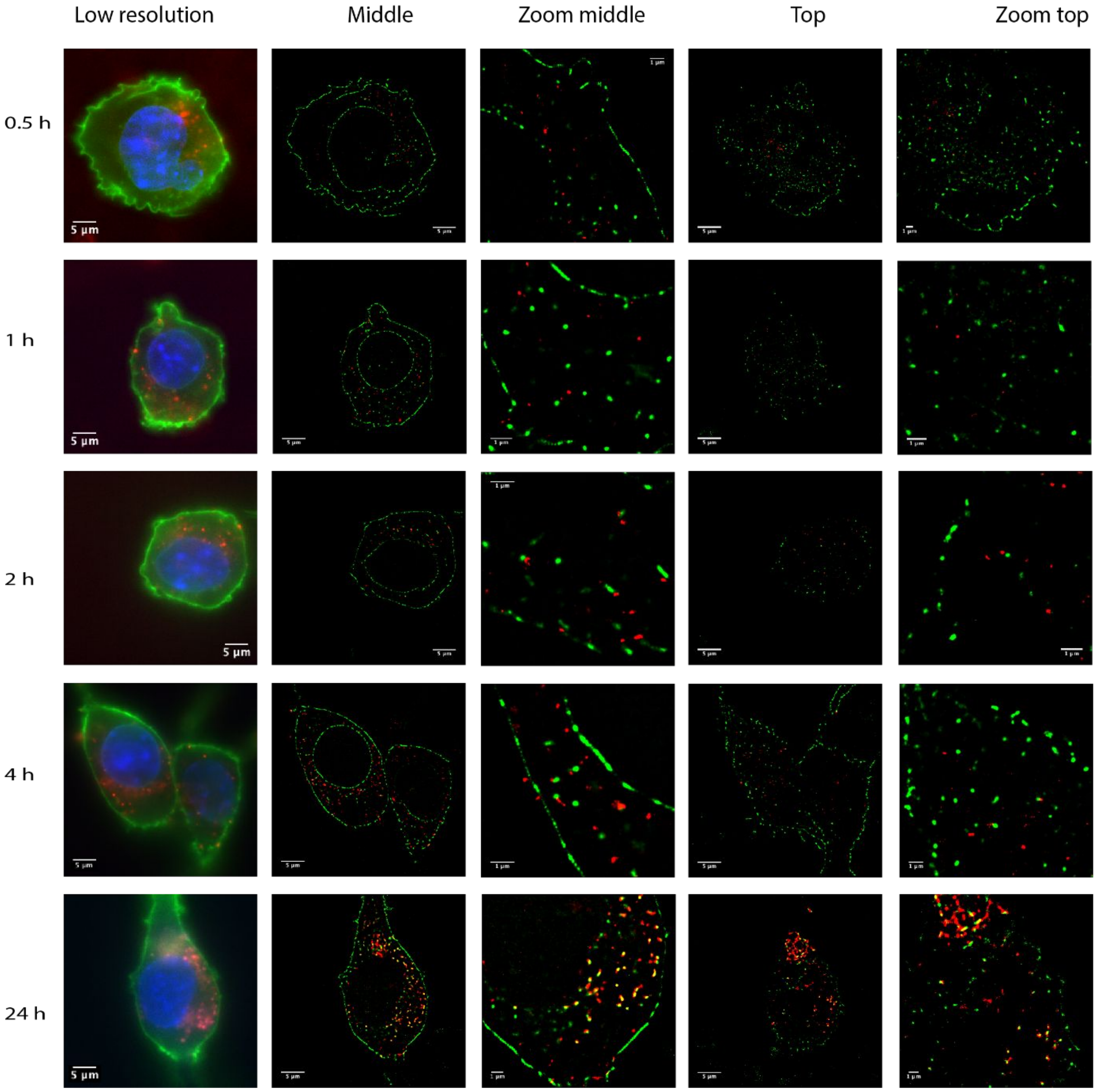

Figure S3 Internalization of the Cy5-GC-CNPs by DC2.4 cell lines at different time points (0.5, 1, 2, 4 and 24h), when incubated with NPs 0.5 µg/ 25,000 cells. The cell membrane is stained with wheat germ agglutinin-488 (WGA-488; green colour), the nucleus stained with DAPI (blue colour) and the nanoparticles are labelled with Cy5 (red colour).

The study on internalization of nanoparticles by DCs by cytometry and laser confocal scanning microscopy (LCSM)

To evaluate the time-dependent uptake of CNPs by human MoDCs, iDCs ($5x10^5$ per well) were plated into a 24-well plate with 0.5 mL of R10 media. Immediately, Cy5-labelled GC-CNPs (Cy5-GC-CNPs) were added at a concentration of 50 µg/mL. At different time intervals (0, 0.5, 1, 2 and 4 h of incubation), cells were collected for uptake analysis. The collected cells were washed immediately with PBS and fixed in a flow cytometry tube using 200 µL of PBS

containing 1% paraformaldehyde (1% PFA). As a control, the MoDCs treated with an equal amount of CNPs at 4 °C was used. The samples were diluted with 500 µL of PBS, and the suspension was analysed by flow cytometry (BD FACS Calibur™ cytometer).

As can be seen in Figure S3, the pattern of GC-CNPs (50 µg/ml) internalization in DCs at 37 °C shows greater uptake with the increase in incubation time. However, only 10% increase in the GC-CNPs uptake was observed between 2 h and 4 h of incubation at 37 °C. While the DCs incubated at 4 °C as a control (treated with 50 µg/ml of GC-CNPs), did not show any significant uptake of GC-CNPs even after 4 h, showing a specific energy-dependent nanoparticle uptake at 37 °C.

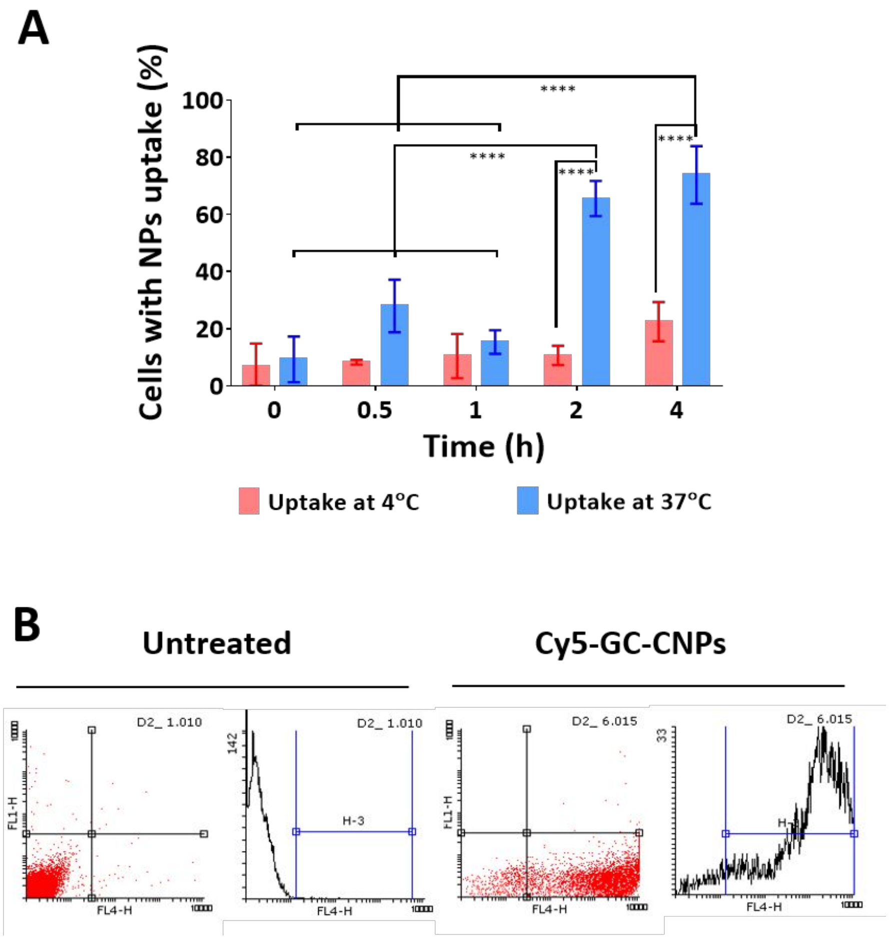

Figure S4: The internalization of the Cy5-GC-CNPs are studied in MoDCs. As shown in **(A)** Time-dependent uptake of Cy5-labeled GC-CNPs (50 µg/ml) at 4 °C and 37 °C analyzed by flow cytometry. **(B)** Comparison of untreated DCs with Cy5-GC-CNPs (50 µg/ml for 2 h at 37 °C) treated DCs, is represented as dot plot and histogram. Results are presented as mean± SD from 3 donors. Statistical difference between the groups is *, $P < 0.05$; **, $P < 0.01$; ***, $P < 0.001$; ****, $P < 0.0001$

For LCSM analysis, the DCs (5×10^5 per well) were plated into a 24-well plate with 0.5 mL of R10 media and incubated for 30 min. Following that the DCs were incubated with the Cy5

labelled GC-CNPs for 1 h. After the incubation time, the DCs were washed with PBS and seeded at a density of 1 x 10^5 onto the poly-L-lysine coated coverslips and allowed to adhere for 15 min. Afterwards, the cells were fixed with 4% PFA for 15 min. The DCs were permeabilized/ fixed using 100 µL of BD Citofix / Citoperm™ (15 min, RT, in the dark) and were washed twice with PBS. The cells were stained using DAPI and phalloidin-488 and fixed on the glass slide using mounting medium (Vectashield® Antifade Mounting Medium, Vector Labs, USA). The image acquisition was made using CLSM (Leica SP5, Mannheim, Germany). Excitation wavelengths were 670 nm for Cy5 and 488 nm for Alexa 488.

In the Figure S5, the co-localization of Cy5-GC-CNPs (red) and the nucleus stain (blue) confirms the uptake of nanoparticles by the DCs. The z-stack of the images in Figure S5A confirms the internalization of the nanoparticles by DCs. Further, cytoskeleton labelling (phalloidin-488, green) of the DCs were performed to see if the nanoparticles were associated with the cell membrane or completely internalized. The Z-stack of the images in the figure 4B confirms that the nanoparticles were mostly inside the DCs with very slight or no adsorption to the surface of DCs.

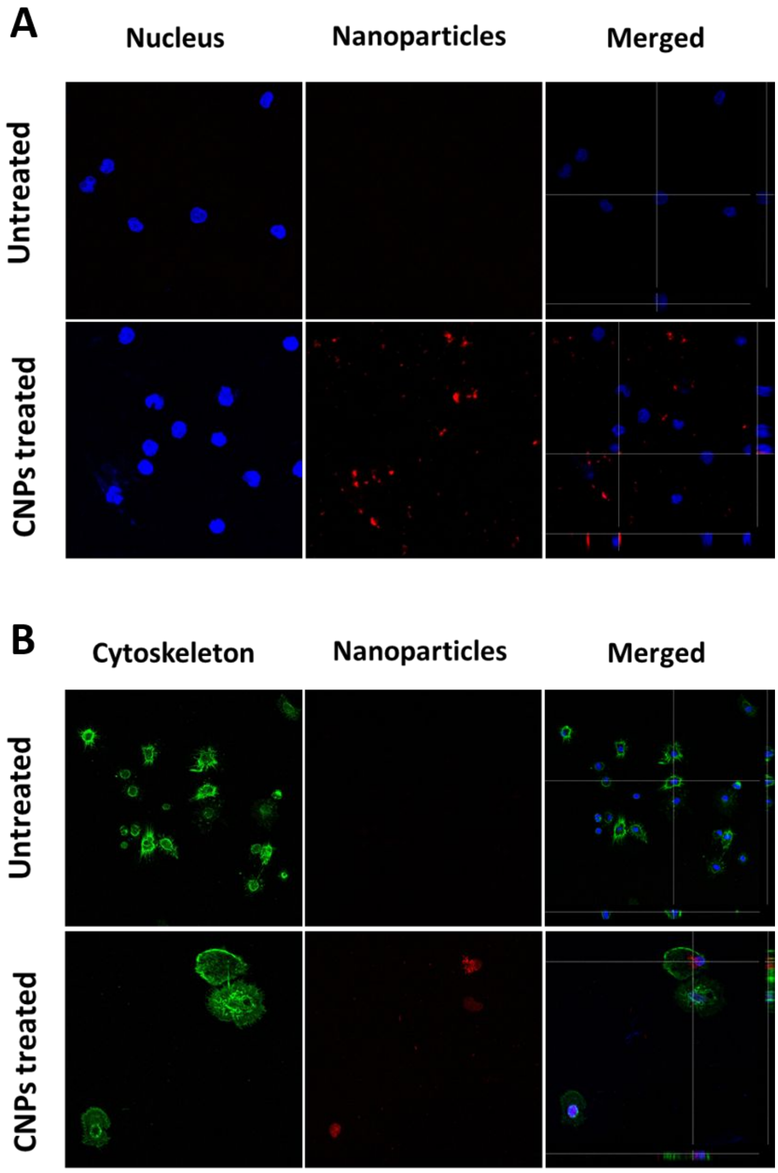

Figure S5: **Internalization of the Cy5-GC-CNPs in DCs.** Confocal microscopy images of the DCs incubated without or with Cy5-GC-CNPs. **(A)** DCs with DAPI staining and **(B)** DCs with cytoskeleton staining using phalloidin-488 (F-actin) and nucleus staining using DAPI. Red channel: Cy5-GC-CNPs; blue channel: cell nuclei; green channel: cytoskeleton.

References

(1) Instructions Pierce LAL Chromogenic Endotoxin Quantitation Kit https://assets.thermofisher.com/TFS-Assets/LSG/manuals/MAN0016039_2162445_Pierce_LAL_Chromo_Endotox_Quant_UG.pdf (accessed 2019 -06 -17).

(2) Brito, L. A.; Singh, M. Acceptable Levels of Endotoxin in Vaccine Formulations During Preclinical Research. *J Pharm Sci* **2010**, *100*, 34–37. https://doi.org/10.1002/jps.22267.
